# Prevalence of smoking and other smoking related behaviors reported by the Global Youth Tobacco Survey (GYTS) in four Peruvian cities

**DOI:** 10.1186/1471-2458-8-S1-S2

**Published:** 2008-12-15

**Authors:** Alfonso Zavaleta, Maria Salas, Armando Peruga, Ana Luiza Curi Hallal, Charles W Warren, Nathan R Jones, Samira Asma

**Affiliations:** 1 Centro de información y educación para la prevención del abuso de drogas (Cedro), Av. Roca y Boloña 271, Lima 18, Peru; 2 Department of Biochemistry, Molecular Biology and Pharmacology, Faculty of Sciences and Philosophy, Peruvian University Cayetano Heredia, Av. Honorio Delgado 430, Lima 30, Peru; 3 Tobacco Free Initiative, World Health Organization, 20 Avenue Appia, 1211 Geneva 27, Switzerland; 4 Tobacco Control and Consumers' Health Team, Pan American Health Organization, 525 23rd St NW, Washington, DC 20037, USA; 5 Office on Smoking and Health, Centers for Disease Control and Prevention, 4770 Buford Highway, Mailstop K-50, Atlanta, Georgia 30341, USA

## Abstract

**Introduction:**

In 2004, Peru ratified the Health Organization (WHO) Framework Convention on Tobacco Control (FCTC) and in 2006 passed Law 28705 for tobacco consumption and exposure reduction. The Global Youth Tobacco Survey (GYTS) provides data on youth tobacco use for development of tobacco control programs. Findings from the GYTS conducted in four main cities in Peru in 2000 and 2003 are reported in this paper and can be used to monitor provisions of the WHO FCTC.

**Methods:**

The GYTS is a school-based survey that uses a standardized methodology for sampling, questionnaire construction, field procedures, and data management. In total, 5,332 and 7,824 students aged 13 to 15 years participated in the 2000 and 2003 surveys conducted in Huancayo, Lima, Tarapoto and Trujillo.

**Results:**

In both years, Lima had the highest lifetime (54.6% and 59.6%) and current use of tobacco (18.6% and 19.2%) of the four cities. According to gender, boys smoked more than girls and less than 20% of students initiated smoking before the age of 10. Among smokers, more than 60% bought their cigarettes in a store with no restriction for their age, and approximately 12% had ever been offered "free cigarettes". Around 90% of students were in favor of banning smoking in public places. Changes between 2000 and 2003 included an increase in the percentage of smokers who wanted to have a cigarette first thing in the morning in Tarapoto (from 0% to 1.2%) and a decrease in exposure to tobacco at home in Huancayo (from 23.7% to 17.8%) and Trujillo (from 27.8% to 19.8%)

**Conclusion:**

While few changes in tobacco use among youth have been observed in the GYTS in Peru, the data in this report can be used as baseline measures for future evaluation efforts. At this time, tobacco control efforts in Peru need to focus on enhancing Law 28705 to include enforcement of existing provisions and inclusion of new laws and regulations. Most of these provisions are required of all countries, such as Peru, that have ratified the WHO FCTC.

## Introduction

The Peruvian government has made tobacco control a priority public health issue. On November 30, 2004, Peru ratified the World Health Organization (WHO) Framework Convention on Tobacco Control (FCTC) [1]. On March 13, 2006, the Peruvian Congress passed Law 28705, "Law of Prevention and Control of Risks Associated with Tobacco Consumption" [2]. Law 28705 entered into effect April 7, 2006. Prior to ratifying the WHO FCTC and passing Law 28705, the tobacco control effort in Peru had been restricted to efforts in individual cities, such as restrictions on the sale of tobacco to minors, and activities on tobacco control promoted in schools [3]. However, regulations on the sale of tobacco products to minors were not enforced and the school programs were not successful as the teachers had not been trained in effective teaching approaches regarding tobacco.

The WHO FCTC was developed as a response to the global tobacco epidemic and is the world's first public health treaty on tobacco control. Countries that ratify the WHO FCTC are encouraged to enact comprehensive tobacco control legislation including, but not limited to: increasing tobacco taxes; protection from exposure to secondhand smoke (SHS); enacting comprehensive bans on tobacco advertising, promotion and sponsorship; and including health warning labels on tobacco packaging. Peru is working to implement laws and regulations to meet the obligations of the WHO FCTC and Law 28705 [2]. However, effective tobacco control policies in Peru must move beyond Law 28705 and focus on new policies related to: increasing taxes on tobacco; banning pro-tobacco advertising and promotion; development of cessation programs; and banning smoking in all indoor workplaces.

Several studies have shown the variations in epidemiological indicators of use of tobacco, and the infrastructure for tobacco control in Peru and other countries of the region [4-8]. In 2002, the annual per capita consumption of tobacco for Peruvians was 1,849 cigarettes [3,9]. In 2003, of the general population (12 to 64 years), more than 75% of people had received an offer to smoke at some time in their life. The life prevalence of tobacco consumption was 63.4% and males consumed more tobacco (83.1%) than females (60.4%) [5,6]. In 2000, the prevalence of tobacco use in youths was 22% for males and 15% for females [10]. The tobacco market in Peru increased from 2001 to 2006, growing at an average annual rate of 4.6% [8].

A model proposed by Lopez and colleagues depicts the global impact of the evolving tobacco pandemic by combining surveillance data on smokers and several indicators of disease burden [4,11]. In stage 1, a country experiences a rather low prevalence of smoking, smoking is essentially limited to males, and there is relatively low disease burden. At present, many of the countries of sub-Saharan Africa are in this stage. Many of the low and middle income countries of Asia, North Africa, and Latin America fit a stage 2 pattern, with persistence of an excess of males among smokers, and with emergence of respiratory complications, cancer, and other tobacco attributable diseases. In stage 3, male and female prevalence estimates converge, sometimes due to a concurrent decline of male smoking and an increase in the proportion of women among active smokers. Many of the countries of southern and eastern Europe are in this stage; deaths attributed to smoking comprise 10% to 30% of all deaths. The more established market economies of northern and western Europe, Australia, and North America are approaching or are in stage 4 of the Lopez and colleagues' model. In this stage, there is a trend over time to a marked decline of smoking incidence and prevalence for both sexes; deaths attributed to smoking peak at 25% to 35% of all deaths, before beginning to decline [4].

The WHO FCTC calls for countries to establish programs for national, regional, and global surveillance. WHO, the US Centers for Disease Control and Prevention, and the Canadian Public Health Association developed the Global Tobacco Surveillance System (GTSS) to assist all 193 WHO member states in establishing tobacco control surveillance and monitoring [12]. The GTSS provides a flexible system that includes common data items but allows countries to include important unique information at their discretion. It also uses a common survey methodology, similar field procedures for data collection, and similar data management and processing techniques. The GTSS includes collection of data through three surveys: the Global Youth Tobacco Survey (GYTS) for youth, and the Global School Personnel Survey and the Global Health Professional Survey for adults. The GYTS has been completed by over 2 million students in 140 countries [13]. In Peru, the GYTS has been implemented in four sites: Huancayo (2000, 2003), Lima (2000, 2003), Tarapoto (2000, 2003) and Trujillo (2000, 2003) [14,15].

The purpose of this paper is to use data from the GYTS conducted in Peru in 2000 and 2003 to set the baseline among youth for self-reported prevalence of tobacco use and smoking cessation, exposure to SHS and tobacco industry marketing, access to and availability of tobacco products, and school curricula teachings.

## Methods

The GYTS is a school-based survey of defined geographic sites that can be countries, provinces, cities, or any other sampling frame, including subnational areas, non-member states, or territories. The GYTS uses a standardized methodology for constructing sampling frames, selecting schools and classes, preparing questionnaires, carrying out field procedures, and processing data. The GYTS questionnaire is self-administered in classrooms, and school, class, and student anonymity is maintained throughout the GYTS process. Country-specific questionnaires consist of a core set of questions that all countries ask and unique country-specific questions. The final country questionnaires are translated in-country into local languages and back-translated to check for accuracy. GYTS country research coordinators conduct focus groups of students aged 13 to 15 years to further test the accuracy of the translation and student understanding of the questions.

The GYTS enquired about several important tobacco-use indicators, including: current cigarette smoking (based on a response of "1 or more days" to the question, "During the past 30 days (1 month), on how many days did you smoke cigarettes?"); current use of tobacco products other than cigarettes; 'susceptibility' (that is, absence of a firm decision not to smoke) or likely initiation of cigarette smoking in the next year among never smokers (based on a negative response to the question, "Have you ever tried or experimented with cigarette smoking, even one or two puffs?" as well as a response of anything but "definitely no" to the questions, "If one of your best friends offered you a cigarette, would you smoke it?" and "Do you think you will try smoking a cigarette in the next year?") [16]; exposure to cigarette smoke in public places (based on a response of "1 or more days" to the question, "During the past 7 days, on how many days have people smoked in your presence, in places other than your home?"); one or more parents smoke cigarettes (based on a response of "both", "father only", or "mother only" to the question, "Do your parents smoke?"); one or more best friends smoke cigarettes (based on a response of "most" or "all" to the question, "Do most or all of your best friends smoke?"); in favor of banning cigarette smoking in public places (based on a positive response to the question, "Are you in favor of banning smoking in public places (such as in restaurants, in buses, streetcars, and trains, in schools, on playgrounds, in gyms and sports arenas, in discos?"); and exposure to pro-tobacco advertising and promotion, either direct or indirect (based on: a response of "a lot" or "a few" to the questions, "During the past 30 days (1 month), how many anti-smoking media messages (for example, television, radio, billboards, posters, newspapers, magazines, movies, drama) have you seen or heard?", "During the past 30 days (1 month), how many advertisements for cigarettes have you seen on billboards?", "During the past 30 days (1 month), how many advertisements for cigarettes have you seen at point of sale?", "During the past 30 days (1 month), how many advertisements or promotions for cigarettes have you seen in newspapers or magazines?"; a positive response to the questions, "Do you have something (t-shirt, pen backpack, etc) with a cigarette brand logo on it?" or "Has a cigarette company representative ever offered you a free cigarette?").

*t*-Tests were used to determine differences between subpopulations [17]. Differences between prevalence estimates were considered statistically significant if the *t*-test *p*-value was < 0.05.

The GYTS uses a two-stage cluster sample design that produces representative samples of students in grades associated with ages 13 to 15 years. The sampling frame includes all schools containing any of the identified grades. At the first stage, the probability of schools being selected is proportional to the number of students enrolled in the specified grades. At the second sampling stage, classes within the selected schools are randomly selected. All students in selected classes attending school the day the survey is administered are eligible to participate. Student participation is voluntary and anonymous using self-administered data-collection procedures. The GYTS sample design produces representative, independent, cross-sectional estimates for each site. For cross-site comparisons, data in this paper are limited to students aged 13 to 15 years old.

A weighting factor is applied to each student record to adjust for non-response (by school, class, and student) and variation in the probability of selection at the school, class, and student levels. A final adjustment sums the weights by grade and gender to the population of school children in the selected grades in each sample site. SUDAAN, a software package for statistical analysis of correlated data, was used to compute standard errors of the estimates and produced 95% confidence intervals, which are shown as lower and upper bounds [18]. Significant statistical differences are noted at the *p *< 0.05 level.

The GYTS was implemented in the same four sites of Peru in 2000 and 2003 using the same methodology and similar sample sizes; 5,332 and 7,824 students in the second to fourth grades of high school participated in each survey, respectively, in Huancayo (1,351 in 2000, 1,923 in 2003), Lima (1,647 in 2000, 1,823 in 2003), Tarapoto (1,057 in 2000, 1,892 in 2003) and Trujillo (1,277 in 2000, 2,186 in 2003). School response rates were greater than 96% in all sites and reached 100% in two sites. The student response rate ranged from 86% to 92% [14,15].

The findings in this report are subject to at least three limitations. First, because the sample surveyed was limited to youths attending school, they may not be representative of all 13 to 15 year olds in Peru. Second, these data apply only to youths who were in school the day the survey was administered and completed the survey. Student response was quite high (86% to 92% in 2000; 89% to 93% in 2003 [14,15]), suggesting bias due to absence or non-response is small. Third, data are based on self reports of students, who may under- or over-report their use of tobacco. The extent of this bias can not be determined in the Peru data; however, responses to tobacco questions on surveys similar to GYTS have shown good test-retest reliability [19].

## Results

### Prevalence

Over 40% of students had ever smoked a cigarette for all four sites in both years (Table 1). In 2003, Huancayo and Lima (58.6% and 59.6%, respectively) had significantly higher prevalences of lifetime cigarette smoking than Trujillo. There was no significant change over time for any of the four sites between 2000 and 2003. Of ever smokers, approximately 1 in 10 in each of the four sites had initiated smoking before age 10, with no significant change between 2000 and 2003.

**Table 1:** Percent of students who had ever smoked cigarettes, ever smoked their first cigarette before age 10, and of students who had never smoked cigarettes that are likely to initiate smoking in the next year (that is, are susceptible), Peru GYTS, 2000 and 2003

**Site**	**Ever smoked cigarettes, even one or two puffs**	**Ever smokers who initiated smoking before age 10**	**Never smokers susceptible to initiate smoking in the next year**
Huancayo, 2000	47.1 (40.9–53.5) (n = 963)	18.1 (13.6–23.6) (n = 429)	29.7 (24.8–35.1) (n = 495)
Lima, 2000	54.6 (49.5–59.6) (n = 1,189)	13.5 (11.1–16.4) (n = 605)	25.4 (20.6–31.0) (n = 545)
Tarapoto, 2000	42.5 (35.6–49.8) (n = 750)	10.9 (7.6–15.3) (n = 305)	19.2 (15.5–23.6) (n = 416)
Trujillo, 2000	46.5 (38.7–54.5) (n = 1,005)	12.3 (9.1–16.4) (n = 429)	25.5 (19.7–32.2) (n = 553)
			
Huancayo, 2003	58.6 (52.9–64.1) (n = 966)	11.6 (8.4–15.7) (n = 550)	25.8 (22.0–29.9) (n = 384)
Lima, 2003	59.6 (52.9–66.0) (n = 998)	13.6 (10.2–17.8) (n = 587)	30.5 (24.2–37.8) (n = 388)
Tarapoto, 2003	48.7 (43.5–54.0) (n = 1,082)	8.3 (5.9–11.6) (n = 505)	19.6 (15.6–24.4) (n = 554)
Trujillo, 2003	44.5 (39.0–50.2) (n = 1,305)	13.1 (9.6–17.7) (n = 585)	22.7 (20.8–24.7) (n = 697)

Values are mean (95% confidence intervals).

A series of questions are used to develop an index of likely initiation of smoking among never smokers (that is, susceptibility). Among never smokers, in 2003, susceptibility ranged from 19.6% (Tarapoto) to 30.5% (Lima) (Table 1). There was no significant change between 2000 and 2003 for any of the sites.

In 2003, current cigarette smoking ranged from 15.3% (Trujillo) to 19.2% (Lima) (Table 2). Current use of tobacco products other than cigarettes ranged from 6.1% (Trujillo) to 7.9% (Lima). Use of other tobacco products was significantly less than cigarette smoking in each of the four sites. Less than 5% of current cigarette smokers reported they feel like having a cigarette first thing in the morning (indicative of cigarette dependency) in each of the four sites.

**Table 2:** Percent of students who were current cigarette smokers, current users of tobacco products other than cigarettes, and current smokers who were dependent on tobacco products, Peru GYTS, 2000 and 2003

**Site**	**Current cigarette smokers**	**Currently used other tobacco products**	**Current cigarette smokers who felt like having a cigarette first thing in the morning**
Huancayo, 2000	15.6 (12.0–20.0) (n = 943)	7.6 (5.7–10.2) (n = 997)	0.9 (0.1–7.1) (n = 115)
Lima, 2000	18.6 (15.2–22.5) (n = 1,120)	6.3 (4.6–8.7) (n = 1,208)	3.4 (1.2–8.9) (n = 146)
Tarapoto, 2000	14.3 (11.2–18.1) (n = 712)	5.6 (3.7–8.2) (n = 757)	0.0 (n = 64)
Trujillo, 2000	16.3 (12.8–20.6) (n = 928)	5.3 (3.6–7.5) (n = 1,016)	3.6 (0.7–16.4) (n = 110)
			
Huancayo, 2003	15.6 (12.9–18.8) (n = 929)	6.3 (4.6–8.7) (n = 993)	0.8 (0.1–5.9) (n = 115)
Lima, 2003	19.2 (15.1–24.0) (n = 972)	7.9 (5.9–10.4) (n = 1,040)	2.6 (0.7–9.2) (n = 144)
Tarapoto, 2003	15.5 (12.4–19.1) (n = 1,039)	6.4 (5.0–8.0) (n = 1,123)	1.2 (0.2–8.4) (n = 106)
Trujillo, 2003	15.3 (11.4–20.2) (n = 1,257)	6.1 (4.5–8.3) (n = 1,340)	3.9 (1.2–12.1) (n = 165)

Values are mean (95% confidence intervals).

There was no significant change over time in prevalence of current cigarette smokers, current users of other tobacco products and the desire to smoke first thing in the morning for any of the four sites between 2000 and 2003, except in Tarapoto, where the prevalence of desiring to smoke first thing in the morning increased. There was no difference between boys and girls in current smoking in any of the cities (Table 3). In all four cities, 15-year-old students were more likely than 13 year olds to be current smokers.

**Table 3:** Percent of students who were current cigarette smokers by gender and age, Peru GYTS, 2000 and 2003

		**Gender**		**Age (years)**		
			
**Site**	**Total**	**Male**	**Female**	**13**	**14**	**15**
Huancayo, 2000	15.6 (12.0–20.0) (n = 943)	20.3 (15.4–26.2) (n = 462)	10.6 (6.6–16.6) (n = 471)	6.9 (4.2–11.2) (n = 291)	13.9 (9.5–20.0) (n = 331)	25.5 (18.9–33.4) (n = 321)
Lima, 2000	18.6 (15.2–22.5) (n = 1120)	20.2 (15.8–25.4) (n = 429)	17.4 (13.3–22.4) (n = 680)	10.6 (6.9–15.9) (n = 354)	17.8 (12.9–24.1) (n = 410)	27.0 (21.5–33.3) (n = 356)
Tarapoto, 2000	14.3 (11.2–18.1) (n = 712)	18.6 (14.5–23.5) (n = 356)	9.8 (6.5–14.7) (n = 349)	8.8 (4.8–15.6) (n = 165)	11.9 (8.6–16.1) (n = 267)	19.6 (13.9–26.8) (n = 280)
Trujillo, 2000	16.3 (12.8–20.6) (n = 928)	22.3 (16.3–29.7) (n = 397)	9.6 (5.0–17.5) (n = 517)	9.7 (6.2–14.8) (n = 292)	18.3 (13.0–25.1) (n = 331)	19.4 (14.6–25.4) (n = 305)
						
Huancayo, 2003	15.6 (12.9–18.8) (n = 929)	17.3 (13.3–22.3) (n = 376)	13.8 (11.0–17.2) (n = 534)	7.4 (4.8–11.3) (n = 319)	20.2 (15.0–26.5) (n = 290)	21.1 (15.8–27.5) (n = 320)
Lima, 2003	19.2 (15.1–24.0) (n = 972)	20.8 (15.3–27.7) (n = 491)	17.0 (13.1–21.7) (n = 472)	11.3 (8.6–14.6) (n = 288)	20.6 (14.8–27.8) (n = 343)	24.7 (18.7–31.8) (n = 341)
Tarapoto, 2003	15.5 (12.4–19.1) (n = 1,039)	20.4 (15.5–26.3) (n = 494)	10.0 (7.5–13.4) (n = 538)	9.0 (5.2–15.1) (n = 351)	15.5 (11.4–20.8) (n = 342)	22.3 (17.2–28.3) (n = 346)
Trujillo, 2003	15.3 (11.4–20.2) (n = 1,257)	21.0 (15.8–27.3) (n = 652)	10.1 (6.6–15.1) (n = 594)	8.6 (5.7–12.7) (n = 378)	15.7 (11.8–20.6) (n = 446)	21.4 (13.7–31.8) (n = 433)

Values are mean (95% confidence intervals).

### Exposure to secondhand smoke

In 2003, exposure to SHS at home was significantly higher in Lima and Tarapoto (25.1% and 27.2%, respectively) than in Huancayo and Trujillo (17.8% and 19.8%, respectively) (Table 4). However, exposure to SHS at home declined between 2000 and 2003 in Huancayo (from 23.7% to 17.8%) and Trujillo (from 27.8% to 19.8%).

**Table 4:** Percent of students exposed to smoke at home, exposed to smoke in public places, and support ban on smoking in public places, Peru GYTS, 2000 and 2003

**Site**	**Exposed to smoking from others at home in the past 7 days**	**Exposed to smoke in public places in the past 7 days**	**Thought smoking should be banned in public places**
Huancayo, 2000	23.7 (20.8–26.9) (n = 997)	34.5 (29.9–39.5) (n = 1,000)	89.3 (86.3–91.7) (n = 997)
Lima, 2000	30.9 (27.8–34.1) (n = 1,209)	44.4 (40.8–48.1) (n = 1,211)	88.2 (85.5–90.5) (n = 1,209)
Tarapoto, 2000	33.0 (30.4–35.7) (n = 762)	39.5 (35.8–43.3) (n = 764)	90.5 (87.0–93.1) (n = 759)
Trujillo, 2000	27.8 (25.1–30.7) (n = 1,017)	40.3 (36.2–44.5) (n = 1,021)	89.8 (86.6–92.4) (n = 1,018)
			
Huancayo, 2003	17.8 (15.4–20.6) (n = 970)	30.2 (27.2–33.4) (n = 967)	86.5 (83.8–88.8) (n = 993)
Lima, 2003	25.1 (22.4–27.9) (n = 985)	41.7 (36.2–47.5) (n = 990)	85.9 (81.3–89.5) (n = 1,030)
Tarapoto, 2003	27.2 (24.2–30.5) (n = 1,051)	35.5 (31.8–39.3) (n = 1,055)	88.7 (86.1–90.8) (n = 1,122)
Trujillo, 2003	19.8 (17.6–22.2) (n = 1,299)	33.1 (29.2–37.2) (n = 1,281)	89.2 (86.6–91.4) (n = 1,331)

Values are mean (95% confidence intervals).

In 2003, exposure to SHS in public places was significantly higher in Lima (41.7%) than in Huancayo (30.2%). There was no significant change over time in exposure to SHS in public places in any of the four sites between 2000 and 2003. Almost 9 in 10 students in each of the four sites thought smoking should be banned in public places. There was no significant change in support of smoke free environments in any of the sites between 2000 and 2003.

### Taught in school about tobacco

Students were asked if, during the past school year in classes, they had been taught about the dangers of tobacco, discussed the reasons why young people smoke, or if they had been taught about the effects of tobacco on their health. Students in Tarapoto were more likely than students in the other sites to have been taught about the dangers of tobacco, to discuss the reasons why people their age smoke, or to have been taught about the effects of tobacco on their health (64.9%, 55.3%, and 50.4%, respectively; Table 5). There was no significant change over time for any of the four sites between 2000 and 2003 in any of these measures.

**Table 5:** Percent of students who were taught the dangers of smoking, discussed reasons why people their age use tobacco, or were taught the effects of using tobacco, Peru GYTS, 2000 and 2003

**Site**	**At school during the past year, taught dangers of smoking tobacco**	**At school during the past year, discussed reasons why people their age smoke**	**At school during the past year, taught about the effects of smoking**
Huancayo, 2000	47.1 (43.1–51.1) (n = 986)	44.9 (39.3–50.7) (n = 999)	38.3 (33.2–43.6) (n = 1,000)
Lima, 2000	42.6 (36.4–49.1) (n = 1,194)	33.9 (29.6–38.6) (n = 1,206)	28.6 (24.8–32.9) (n = 1,205)
Tarapoto, 2000	67.1 (59.8–73.7) (n = 761)	52.4 (46.5–58.3) (n = 763)	50.6 (44.5–56.7) (n = 754)
Trujillo, 2000	58.0 (52.6–63.2) (n = 1,008)	49.3 (44.5–54.1) (n = 1,025)	43.5 (37.0–50.1) (n = 1,014)
			
Huancayo, 2003	48.8 (45.3–52.4) (n = 990)	42.8 (39.2–46.4) (n = 993)	36.1 (32.0–40.5) (n = 994)
Lima, 2003	43.2 (37.2–49.3) (n = 1,027)	37.1 (30.7–44.1) (n = 1,040)	32.2 (26.7–38.4) (n = 1,035)
Tarapoto, 2003	64.9 (60.1–69.4) (n = 1,125)	55.3 (51.2–59.4) (n = 1,122)	50.4 (45.5–55.4) (n = 1,123)
Trujillo, 2003	56.6 (51.3–61.8) (n = 1,344)	49.6 (43.7–55.5) (n = 1,347)	43.2 (39.7–46.8) (n = 1,340)

Values are mean (95% confidence intervals).

### Media and advertising exposure

Over 7 in 10 students in each of the four sites reported that they saw tobacco advertisements on billboards during the month prior to the survey (Table 6). A similar proportion reported seeing advertisements for cigarettes in newspapers or magazines in the month prior to the survey, with Lima showing the highest proportion (82.0%) in 2003, a significantly higher exposure than in Huancayo. Approximately 1 in 10 students reported that they had an object (that is, hat, t-shirt, knapsack, and so on) with a cigarette logo on it (Table 6).

**Table 6:** Percent of students who saw advertisements on billboards, or in newspapers or magazines, and had an object with a tobacco company logo on it, Peru GYTS, 2000 and 2003

**Site**	**Saw ads for cigarettes on billboards in the past month**	**Saw ads for cigarettes in newspapers or magazines in the past month**	**Had an object with a cigarette brand logo on it**
Huancayo, 2000	70.3 (65.3–74.8) (n = 989)	77.1 (73.6–80.2) (n = 983)	12.8 (9.8–16.4) (n = 983)
Lima, 2000	78.3 (74.6–81.6) (n = 1,198)	84.7 (82.4–86.7) (n = 1,197)	13.8 (11.4–16.7) (n = 1,190)
Tarapoto, 2000	76.6 (74.1–78.8) (n = 755)	82.5 (79.0–85.5) (n = 752)	7.4 (5.5–9.9) (n = 756)
Trujillo, 2000	71.4 (68.0–74.7) (n = 1,008)	77.6 (72.6–81.8) (n = 1,000)	11.8 (9.0–15.3) (n = 1,003)
			
Huancayo, 2003	69.0 (64.6–73.0) (n = 984)	72.4 (67.4–77.0) (n = 975)	13.7 (11.4–16.4) (n = 965)
Lima, 2003	76.9 (71.5–81.5) (n = 1,027)	82.0 (78.5–85.1) (n = 1,022)	12.5 (9.9–15.8) (n = 1,016)
Tarapoto, 2003	74.4 (70.9–77.6) (n = 1,119)	78.4 (74.8–81.7) (n = 1,116)	10.6 (8.8–12.7) (n = 1,108)
Trujillo, 2003	73.1 (69.3–76.6) (n = 1,328)	78.8 (75.0–82.1) (n = 1,325)	11.7 (9.3–14.6) (n = 1,319)

Values are mean (95% confidence intervals).

### Cessation

In 2003, approximately 7 in 10 students who currently smoked cigarettes in Huancayo, Tarapoto, and Trujillo, and 6 in 10 in Lima, reported that they wanted to stop smoking at the time of the survey (Table 7). A similar proportion of smokers had tried to stop smoking during the past year but failed. The majority of students in each of the four sites who currently smoked at the time of the survey reported that they had received help to quit smoking, with the percent in Tarapoto (75.2%) being significantly higher than in Lima (58.6%). There was no significant change over time in intention to quit or success in quitting in any of the sites between 2000 and 2003.

**Table 7:** Percent of current smokers, current smokers who wanted to quit, who tried to quit, or who received help to quit, Peru GYTS, 2000 and 2003

**Site**	**Current smokers**	**Current cigarette smokers who wanted to stop smoking now**	**Current cigarette smokers who tried to stop smoking during the past year**	**Current smokers who have ever received help to stop smoking**
Huancayo, 2000	15.6 (12.0–20.0) (n = 943)	69.3 (61.4–76.1) (n = 94)	68.1 (59.1–75.9) (n = 96)	64.0 (54.9–72.1) (n = 364)
Lima, 2000	18.6 (15.2–22.5) (n = 1120)	62.0 (51.1–71.8) (n = 102)	61.6 (52.4–70.1) (n = 119)	53.3 (47.4–59.1) (n = 509)
Tarapoto, 2000	14.3 (11.2–18.1) (n = 712)	84.2 (71.8–91.7) (n = 51)	79.5 (68.2–87.6) (n = 56)	70.6 (62.7–77.4) (n = 257)
Trujillo, 2000	16.3 (12.8–20.6) (n = 928)	79.5 (65.1–88.9) (n = 73)	78.7 (65.9–87.6) (n = 84)	71.5 (65.5–76.8) (n = 388)
				
Huancayo, 2003	15.6 (12.9–18.8) (n = 929)	76.2 (67.4–83.2) (n = 76)	75.0 (61.8–84.8) (n = 78)	71.8 (59.2–81.7) (n = 141)
Lima, 2003	19.2 (15.1–24.0) (n = 972)	62.2 (52.4–71.2) (n = 104)	64.5 (52.8–74.6) (n = 107)	58.6 (52.0–64.9) (n = 180)
Tarapoto, 2003	15.5 (12.4–19.1) (n = 1,039)	77.6 (65.1–86.5) (n = 82)	80.5 (66.4–89.6) (n = 80)	75.2 (66.9–81.9) (n = 151)
Trujillo, 2003	15.3 (11.4–20.2) (n = 1,257)	72.8 (63.2–80.6) (n = 124)	73.0 (66.4–78.7) (n = 123)	64.5 (58.5–70.2) (n = 200)

Values are mean (95% confidence intervals).

### Access and availability

Almost 6 in 10 students in each of the four sites who currently smoked in 2003 reported that they "usually" bought their cigarettes in a store (Table 8). There was no significant difference between the sites. Over 7 in 10 current smokers who usually bought their tobacco in a store reported they had not been refused purchase because of their age in all the sites. In addition, approximately 1 in 10 students in each of the four sites had been offered "free" cigarettes by tobacco company representatives.

**Table 8:** Percent of current smokers who usually bought cigarettes in a store, of those who bought in a store the percent not refused purchase because of their age, and those offered free cigarettes by a tobacco company representative, Peru GYTS, 2000 and 2003

**Site**	**Current smokers who usually bought their cigarettes in a store**	**Current smokers who usually bought their cigarettes in a store who were not refused purchase because of their age**	**Ever been offered "free" cigarettes by a cigarette company representative**
Huancayo, 2000	59.3 (49.8–68.2) (n = 146)	89.8 (79.4–95.3) (n = 81)	11.3 (9.0–14.0) (n = 991)
Lima, 2000	62.4 (55.9–68.5) (n = 194)	70.3 (60.6–78.5) (n = 96)	9.4 (7.9–11.2) (n = 1,201)
Tarapoto, 2000	53.6 (42.9–64.1) (n = 95)	75.2 (59.2–86.4) (n = 43)	8.1 (6.2–10.5) (n = 760)
Trujillo, 2000	59.9 (50.9–68.3) (n = 141)	87.2 (78.4–92.7) (n = 73)	9.6 (7.5–12.2) (n = 1,012)
			
Huancayo, 2003	66.2 (56.3–74.8) (n = 143)	72.0 (63.0–79.6) (n = 83)	11.2 (8.9–14.1) (n = 978)
Lima, 2003	59.3 (50.3–67.6) (n = 189)	73.9 (62.3–82.9) (n = 97)	9.7 (7.3–12.8) (n = 1,028)
Tarapoto, 2003	53.9 (46.6–61.1) (n = 151)	77.2 (64.8–86.1) (n = 68)	8.9 (7.0–11.4) (n = 1,117)
Trujillo, 2003	71.0 (64.1–77.0) (n = 207)	70.1 (60.9–78.0) (n = 131)	7.7 (6.0–9.7) (n = 1,326)

Values are mean (95% confidence intervals).

## Discussion

WHO FCTC Article 20 calls for countries to use consistent methods and procedures in their tobacco control surveillance efforts [1]. All GYTS surveys use exactly the same sampling procedures, core questionnaire items, training protocol, and field procedures [20-22]. Therefore, the analysis of data is consistent and comparable across all survey sites and over time. The data in this report show that for 13 to 15 year olds in four cities in Peru, approximately 15% currently smoked cigarettes and approximately 6% used other tobacco products. There was no change between 2000 and 2003 in the prevalence of tobacco use. The results from these surveys can be used to set a baseline for monitoring specific tobacco control interventions in Peru as they relate to WHO FCTC articles and Peruvian Law 28705 and further enhance the capacity of the country to develop, implement, and evaluate their tobacco control programs.

Over 3 in 10 students reported that they were exposed to smoke in public places. Article 8 of the WHO FCTC addresses the need to protect the population from exposure to tobacco smoke and Peruvian Law 28705 completely bans smoking in public and private establishments dedicated to health and education. However, Law 28705 only partially restricts smoking in workplaces, hotels, restaurants, coffee shops, bars and other recreational centers, allowing designated smoking areas [2]. The GYTS results show that between 2000 and 2003 the percentage of students exposed to SHS in public places did not change, but that more than 80% of respondents thought that smoking should be banned in all public places. In Peru, the 2003 GYTS was applied in schools between September and November, and exposure to SHS at home, in the week prior to the survey, decreased in Huancayo and Trujillo. This could be related to smoke free campaigns led by non-governmental organizations for the approval and ratification of the WHO FCTC in Peru. The existing provisions of Law 28705 must be enforced and it is possible, if presented, that the findings from the GYTS could convince the Peruvian congress to consider amending Law 28705 to include all public workplaces becoming smoke free and to ensure effective enforcement.

Exposure to tobacco advertising is high in Peru and did not change between 2000 and 2003. Nearly 70% of the students in all sites in 2000 and 2003 had seen pro-tobacco advertising on billboards, or in newspapers or magazines in the past month. It is urgent that Article 13 of the WHO FCTC requiring a comprehensive ban on all tobacco advertising, promotion and sponsorship be implemented in Peru. Peruvian Law 28705 prohibits direct and indirect advertising and sponsorship promoting tobacco products in electronic media and all sports events and also prohibits sponsorship by the tobacco industry of events for minors. However, Law 28705 includes only partial restrictions; thus, it is not as strict as Article 13 of the WHO FCTC requires. Studies have shown that the impact of partial restrictions, such as those outlined by Law 28705, have very little effect on the consumption of tobacco [23].

Renewed effort needs to be made to achieve the objectives set by Article 12 of the WHO FCTC on education, communication, training and public awareness and Article 5 in Peruvian Law 28705 dedicated to promote and strengthen public awareness of tobacco control issues. However, education programs will be most effective if they occur after a favorable tobacco control policy environment has been established [23]. Thus, the initial strategy should be to focus on: policies aimed at reducing tobacco consumption, such as increased taxes and prices; 100% smoke free environments in all public places and workplaces; and a comprehensive ban of all tobacco advertising, promotion and sponsorship. Creating this favorable policy environment first is especially important for school programs on preventing tobacco consumption, given the results of a recent literature review showing their ineffectiveness [24] and according to tobacco control policies recommended by WHO [23].

Data in this report further show that over 6 in 10 current smokers want to stop smoking and over 6 in 10 have tried to stop during the past year but have failed. These findings suggest the need to develop, pilot test, and evaluate potential youth cessation programs in accordance with Article 14 of the WHO FCTC and Article 5 of Peruvian Law 28705.

## Conclusion

Between 2000 and 2003, many of the main indicators of the GYTS did not change, given that minimal policies were implemented in Peru during this time [3,23]. With the ratification of the WHO FCTC and the passing of Law 28705, however, new hope has been raised. Development of an effective comprehensive tobacco control program, including population-based intervention efforts to reduce tobacco use – such as smoke-free environment policies, increases in the price of tobacco products, laws that regulate and enforce bans on sales, purchases, and consumption of tobacco products by underage youth, laws that regulate content, labeling, promotion, and advertising of tobacco products, and mass media campaigns – will require careful monitoring and evaluation of existing programs and the likely development of new efforts [25,26]. The synergy between the Peruvian leadership in ratifying the WHO FCTC and in supporting the conduct of the GYTS throughout the country offers

Peru a unique opportunity to develop, implement and evaluate comprehensive tobacco control policies that can lead to a reduction in tobacco consumption, especially among adolescents. Repeating the GYTS in the future will provide data to assess whether the tobacco control policies in Law 28705 are being implemented and enforced.

## List of abbreviations used

FCTC: Framework Convention on Tobacco Control; GTSS: Global Tobacco Surveillance System; GYTS: Global Youth Tobacco Survey; SHS: secondhand smoke; WHO: World Health Organization.

## Competing interests

The authors declare that they have no competing interests.

## Authors' contributions

CWW, NRJ and SA conceived of the study, participated in its design and coordination, and helped to draft the manuscript. AZ was responsible for national coordination of the study and drafted the manuscript. MS, AP and ACH participated in interpretation of the statistical analysis and drafting of the manuscript. All authors read and approved the final manuscript.
